# Calcium-Binding Proteins with Disordered Structure and Their Role in Secretion, Storage, and Cellular Signaling

**DOI:** 10.3390/biom8020042

**Published:** 2018-06-19

**Authors:** Ewa A. Grzybowska

**Affiliations:** Maria Sklodowska-Curie Memorial Cancer Center and Institute of Oncology, Roentgena 5, 02-781 Warsaw, Poland; ewag@coi.waw.pl

**Keywords:** calcium binding proteins, intrinsic disorder, calcium signaling

## Abstract

Calcium is one of the most important second messengers and its intracellular signaling regulates many aspects of cell physiology. Calcium ions, like phosphate ions, are highly charged and thus are able to alter protein conformation upon binding; thereby they constitute key factors in signal transduction. One of the most common calcium-binding structural motifs is the EF-hand, a well-defined helix-loop-helix structural domain, present in many calcium-binding proteins (CBPs). Nonetheless, some CBPs contain non-canonical, disordered motifs, which usually bind calcium with high capacity and low affinity, and which represent a subset of proteins with specific functions, but these functions rarely involve signaling. When compared with phosphorylation-mediated signal transduction, the role of intrinsic disorder in calcium signaling is significantly less prominent and not direct. The list of known examples of intrinsically disordered CBPs is relatively short and the disorder in these examples seems to be linked to secretion and storage. Calcium-sensitive phosphatase calcineurin is an exception, but it represents an example of transient disorder, which is, nevertheless, vital to the functioning of this protein. The underlying reason for the different role of disordered proteins in the two main cellular signaling systems appears to be linked to the gradient of calcium concentration, present in all living cells.

## 1. Calcium Signaling in the Cell

In order to survive and function, cells must properly recognize and respond to various extracellular stimuli. Such stimuli are primarily perceived by the first messengers: i.e., hormones, growth factors, or neurotransmitters. First messengers do not enter the cell, but, by binding to specific receptors, activate a signaling cascade inside the cell. This signaling is mediated by the second messengers: cAMP, cGMP, inositol trisphosphate, diacylglycerol, and calcium. Ca^2+^ ions are particularly well suited for signaling purposes, because cells strictly maintain a 20,000- to 100,000-fold gradient between their intracellular (cytoplasmic) and extracellular concentration [1]. The Ca^2+^ concentration is also high in specific intracellular compartments, like endoplasmic reticulum and mitochondria, which thereby function as calcium stores, with the potential to open gates and generate calcium flux. Keeping calcium out of the cytoplasm is vital, because calcium ions are potentially dangerous. They have the ability to precipitate both inorganic and organic anions, including phosphate, possibly incapacitating phosphate-based energy metabolism [2,3]. To prevent this, Ca^2+^ must be chelated, sequestered, and stored, which is achieved by constantly operating multiple calcium pumps and the appropriate calcium-buffering and sequestering factors. Magnesium is a similar cation, but without such detrimental properties, since it binds water more tightly and its concentration gradient is practically negligible. The steep Ca^2+^ concentration gradient provides a golden opportunity for signal transduction, and cells exploit it. Every change in Ca^2+^ concentration can be easily detected and propagated. Calcium signaling tends to be rapid, localized, and propagated as spikes, waves, or oscillations [4]. A web of endoplasmic reticulum permeates the entire cell, which enables local channel-opening and compartmentalization of the signal. In excitable cells (myocytes, neurons), these signals propagate rapidly, while in the epithelial cell layer, intercellular transmission is slower and occurs mainly through gap junctions [4].

This type of signaling regulates various physiological processes, like fertilization, embryonic pattern formation, differentiation, proliferation, apoptosis, learning and memory, muscle contraction, and secretion [5].

## 2. Calcium-Binding Proteins

Calcium-binding proteins (CBPs) not only control cytoplasmic Ca^2+^ concentration—by means of a multiple calcium pumps, channels, sequestering agents, and buffers—but also function as Ca^2+^ transporters and calcium-sensors with regulatory potential, including transcription factors and enzymes, which elicit the appropriate response to calcium fluxes [1]. The most prevalent and studied CBPs are those bearing the EF-hand domain, like parvalbumin, calmodulin, S100 proteins, and calcineurin. CBPs with an ordered structure, but lacking EF-hand, include proteins with a C2 domain (protein kinase C, PKC), an EGF-domain (epidermal growth factor), a GLA (γ-carboxyglutamic) domain (osteocalcin, matrix GLA protein, periostin) and other domains (reviewed by Yanez et al., Kirberger and Yang [6,7]). The most prevalent, versatile, and ubiquitous CBP, with a plethora of cellular targets, is calmodulin (calcium-modulated protein, CaM), the universal calcium sensor, which regulates many cellular pathways. CaM itself is well structured, but, as discussed in detail below, binds to many intrinsically disordered proteins.

## 3. The Regulatory Potential of Intrinsically Disordered Proteins

The most important feature of intrinsically disordered proteins (IDPs) is the absence of an ordered three-dimensional structure. A protein is classified as an IDP if it possesses a region of at least 30 amino acids (aa), which is unstructured under physiological conditions. Such an unstructured region has a great regulatory potential, since it can fold upon the ligand binding (the ligand-induced disorder-to-order transition), and this conformational change usually entails some kind of signal transduction [8,9,10]. Alternatively, disordered proteins may never adopt any specific structure and alternate between several possible conformations. Complexes of such proteins are termed “fuzzy complexes”, analogous to “fuzzy logic” in mathematics. This offers a great regulatory capacity, for example, it enables a more exact, fine-tuned response, when some motif (e.g., leucin-dependent endocytosis motif or phosphorylation site) is only transiently available. 

Intrinsically disordered proteins are quite numerous, especially in more complex organisms: IDP coding genes constitute about 4% of the bacterial genomes (2% in Archea), 33% of eukaryotic genomes, and almost half of all protein-coding genes in the human genome (44%) [11]. Such findings contributed to a paradigm shift in protein structure-to-function relationship over the last two decades [12]. Moreover, IDPs are overrepresented within regulatory proteins and often constitute “hubs” which can potentially regulate entire signaling pathways. Of particular interest is the large number of IDPs that undergo phosphorylation, sometimes very intensely. *Neurospora* protein Frequency (FRQ) bears as much as 113 phosphorylation sites, with phosphorylation occurring sequentially during a day, thereby enabling a molecular clock-like action [13]. Multiple phosphorylation sites are often located in poorly structured regions, enabling a conformational switch, upon accumulating sufficient negative charge. There are many examples of phosphorylation-dependent molecular switches, often coupled with degradation (phospho-degron: Sic1 [14], Eco1, [15]).

## 4. Calcium-Binding IDPs

Hundreds of cellular proteins are able to bind calcium at affinities ranging widely from the nM to mM scale, but only a fraction of them can be classified as IDPs. This is in stark contrast to the multiple IDPs involved in phosphorylation, the other most common cellular signaling system. The calcium-binding IDPs known to date constitute a very specific set of proteins, these include one type of bacterial toxins, proteins from various taxa involved in biomineralization, calmodulin-binding protein calcineurin, sarcoplasmic/endoplasmic reticulum proteins involved in calcium storage, and some other poorly characterized examples, without any obvious affiliation. In this review, this short list of calcium-binding IDPs will be analyzed to find a common thread and address the apparent difference in IDPs usage in cellular signal transduction systems.

### 4.1. Bacterial Calcium-Binding Repeat-in-Toxin Motifs

**Figure 1:**
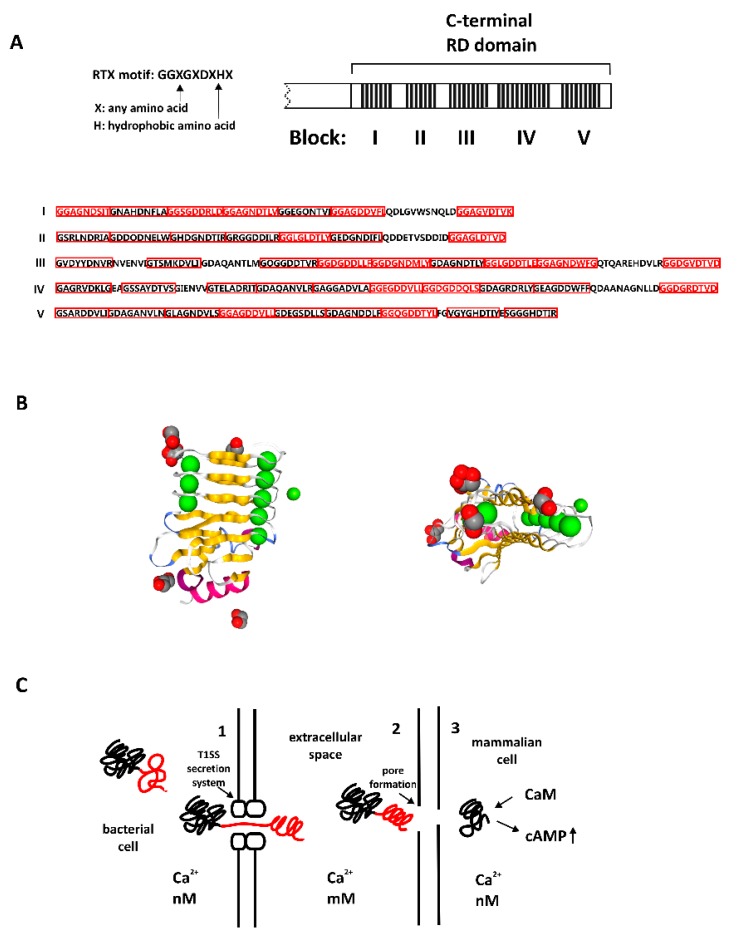
Structure and function of bacterial adenylate cyclase toxin (CyaA). (**A**) Structure of the C-terminal RTX domain (RD) of CyaA, with five blocks containing repeat-in-toxin (RTX) consensus. Variability of the motif can be observed in all blocks, RTX motifs in red frames, full consensus in red letters. (**B**) Left panel: calcium-bound RTX domain of bacterial toxin CyaA, β-roll structure, PDB_5CVW, right panel: the same structure from another perspective, visible narrow parallel β-roll. (**C**) Bacterial toxin with RTX domain acquires the structure upon Ca^2+^ binding. **1**. Protein in bacterial cell: C-terminal domain is unstructured (red) in a low Ca^2+^ concentration. **2**. Protein translocates into extracellular space through type 1 secretion system (T1SS): in a high concentration of calcium ions C-terminal domain acquires secondary structure (β-roll) **3**. The toxin forms pores in mammalian cell membrane; part of the toxin (catalytic domain) translocates into the mammalian cell, binds calmodulin and causes detrimental increase in cellular cAMP.

Repeat-in-toxin (RTX) motifs were found in over 250 toxins and other proteins secreted by gram-negative bacteria [16]. RTX proteins perform diverse biological functions; proteins belonging to this family encompass pore-forming toxins (leukotoxins, hemolysins), metalloproteases and lipases, involved in virulence and invasion of animal tissues, but also bacterial toxins (bacteriocins) that act against other bacterial strains. Other members of this family include surface-layer (S-layer) forming proteins, involved in providing bacterial cell protection and motility and proteins involved in nitrogen fixation (nodulation) in the symbiotic bacteria of higher plants [17]. The adenylate cyclase toxin (CyaA) from *Bordetella pertussis*, an agent responsible for whooping cough, has been the most widely investigated example. This large protein is composed of five regions, consisting of an adenylate cyclase catalytic domain (ACD), a translocation region (TR), a hydrophobic region (HR), an acylation region (AR), and a C-terminal RTX domain (RD), containing many RTX repeats (Figure 1A). It has been shown that the RTX region of this protein exhibits intrinsic disorder in the absence of calcium [16,18].

RTX proteins differ in size and function, but all possess two crucial features: the RTX sequence repeats and that they all are secreted via the type 1 secretion system (T1SS). The number of RTX sequence repeats vary between 5 to 50 [2]. The RTX motif comprises glycine and aspartate rich nonapeptides (Figure 1A), which confer negative charge and cause electrostatic repulsion between residues, contributing to the disorder. Ca^2+^ binding neutralizes the charge and induces a disorder-to-order transition, promoting folding and the formation of the characteristic super secondary structure termed the parallel β-roll or the parallel β-helix (Figure 1B, NGL viewer [19]). Calcium ions bind to both sides of the structure, affecting the angle of twist, contributing to the formation of its narrow hydrophobic core. One turn of this helix contains two RTX motifs [16]. Calcium binding causes compaction, folding, and stabilization of the structure of the otherwise disordered regions.

A low Ca^2+^ concentration within bacterial cells prevents RTX proteins from folding, which is triggered by protein secretion into the extracellular space. This is accomplished by the T1SS. Notably, many RTX proteins are large (e.g., CyaA is about 180 kDa), so the translocation is quite difficult and requires energy. The C-terminal secretion signal of the RTX protein is recognized by the ATP-ase driven inner-membrane complex, which contacts outer membrane proteins, assembling a channel spanning the entire cell envelope, so that proteins can be secreted directly into the extracellular space. Transfer to the millimolar calcium concentration promotes the loading of the RTX repeats with calcium ions and acquisition of the structure. This transfer occurs starting from the C-terminus, which often bears RTX repeats or a RTX domain, as in the CyaA protein. Imminent folding and stabilization of the protein within extracellular space has even been proposed to facilitate the secretion process [16]. Thus, for RTX proteins, folding depends on protein secretion, but it may also contribute to it.

For those RTX proteins that are toxins, the next step is to bind to the receptors on the plasma membrane of the host cell, form a pore, and translocate its catalytic domain directly to the host cytoplasm. All these activities require calcium and an ordered, calcium-induced RTX structure. The translocated catalytic domain becomes activated by endogenous calmodulin, resulting in a significantly elevated cAMP concentration, which is detrimental to host cell physiology. Furthermore, pore formation induces calcium influx into target cells, disrupting calcium homeostasis and contributing to the toxicity [16] (Figure 1C).

### 4.2. Disordered Calcium-Binding IDPs in Biomineralization

There is a large group of calcium-binding disordered proteins involved in biomineralization. Significantly, structure prediction analyzes indicate that almost all proteins involved in biomineralization in the Swiss Protein Database are intrinsically disordered [20]. Biomineralization is a process where mineral materials are deposited in living organisms, giving rise to inorganic-based skeletal structures such as bone and dentine along with exoskeletal structures like shells, carapaces, or coral scaffolds. Calcium is an essential component of these structures, mainly in the form of calcium carbonate or calcium phosphate. As a result of biomineralization, calcium carbonate comprises more than 4% of the earth’s crust and is commonly found in rocks of organic origin [1]. Biologically controlled biomineralization is complex and is orchestrated in space and time by concentration (to supersaturation), initiation of mineral formation, secretion, and deposition of organic matrix molecules onto a specific surface [21]. Living cells accomplish this by using a group of specific proteins which facilitate the initial crystal formation (i.e., a critical nucleus) from amorphous aggregates and then control the deposition. The deposition must be controlled, to prevent any rapid and excessive precipitation. During bone formation, the deposition of organic matrix occurs onto collagen matrices, where minerals are delivered by extracellular vesicles (matrix vesicles, MV), small (20–200 nm) membrane structures, which bud off from the plasma membrane of chondrocytes, osteoblasts, and odontoblasts [22,23]. The calcium-binding proteins involved in biomineralization usually have an acidic domain, which both attracts positively charged calcium ions, and causes a repulsion due to the negative charge, leading to structural disorder [20]. Unlike the case of the RTX toxin, Ca^2+^ binding does not often force structure formation, thereby underlying the importance of the disorder to the process of biomineralization.

The best characterized examples of the calcium-binding IDPs involved in biomineralization are described below (summarized in Table 1).

#### 4.2.1. Bone and Dentin Biomineralization

Small integrin-binding ligand N-linked glycoproteins (SIBLINGs) is a family of secreted, extracellular proteins involved in the biomineralization of bones and teeth [24,25]. They share common features, like the RGD (arginine-glycine-aspartate) motifs responsible for the interaction with integrins, acidity (especially after phosphorylation), intrinsic disorder, extensive post-translational modifications (phosphorylation, glycosylation), and calcium-binding and collagen-binding properties [21,24,25]. Proteins belonging here include: osteopontin (OPN), dentin matrix protein 1 (DMP1), matrix extracellular phosphoglycoprotein (MEPE), bone sialoprotein (BSP), and dentin sialophosphoprotein (DSPP); the latter being proteolytically processed to dentin phosphophoryn (DPP) and dentin sialoprotein (DSP).

Interestingly, the hardest mineral in vertebrates, tooth enamel, is formed by epithelial secretion, in contrast to bone and dentin, which are produced by the mesenchymal extracellular matrix. Amylogenin, which constitutes the majority of immature enamel, undergoes extracellular self-assembly that requires calcium and phosphate. It can act both as an extracellular scaffold for mineral crystal formation and as a factor controlling the direction of crystal growth [20].

#### 4.2.2. Fish Otolith Formation

Searching through zebrafish cDNA libraries for *dspp*-like genes helped in the discovery of yet another group of calcium-binding IDPs involved in biomineralization: proteins that control fish otolith formation [26]. An otolith (also called stratolith or otoconia in land vertebrates) is a calcium carbonate structure in the inner ear of vertebrates, involved in hearing and balance. To date, the best described examples of the calcium-binding IDPs involved in otolith formation include the Starmaker protein (Stm) of zebrafish (*Danio rerio*) [27], and its putative functional analog otolith matrix macromolecule-64 (in rainbow trout) [28]. Stm regulates otolith formation in a concentration-dependent manner. Phosphorylation of Stm increases its calcium-binding ability and changes the dissociation constant [27]. Calcium ion binding induces compaction of the Stm molecule, causing a significant decrease in its hydrodynamic radius and inducing secondary structure formation [29]. Similarly, otolith matrix macromolecule-64 (OMM-64) also displays molecular compaction upon calcium binding, but it remains disordered [28].

#### 4.2.3. The Role of Calcium in Biomineralization and Disorder

It should be pointed out that although IDPs are over-represented among the proteins involved in biomineralization, calcium-binding proteins constitute only a subset, and only in specific cases do they attain a secondary structure due to calcium-binding. Thus, there appears to be an open question: is calcium binding inherently significant to the structural disorder of the IDPs involved in biomineralization, or rather, is this property coupled to the role of calcium as a transported cargo? The presence of calcium-binding acidic domains certainly promotes disorder. A substantial proportion of these proteins also undergo phosphorylation. It has been hypothesized that posttranslational modifications like phosphorylation and glycosylation, coupled with surface-binding, can stabilize the structure of an IDP. There is a possibility that the free energy required for surface adherence to initiate mineral deposition may be lower for IDPs caused by a change in entropy during the disorder-to-order transition [21]. This however only addresses the question of why so many IDPs are involved in biomineralization, but not specifically the calcium-binding subset.

An interesting example of IDPs linked to biomineralization are caseins; unfolded, extremely flexible, and very abundant milk proteins. Caseins possess the ability to assemble colloidal protein particles, casein micelles, which can sequester and transport amorphous calcium phosphate [30,31]. It has been hypothesized, that caseins (and other IDPs) may have a kinetic advantage over other proteins, owing to their flexible structure and relatively large surface area, accelerating precipitation from supersaturated solution [20].

### 4.3. Calmodulin-Binding IDPs

**Figure 2:**
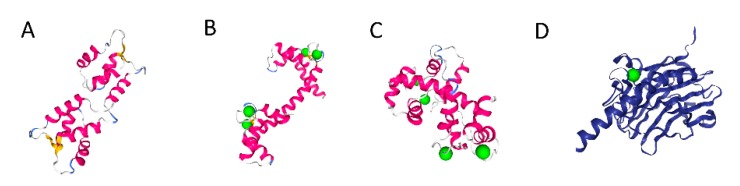
Ca^2+^ binding by structured motifs of calmodulin and calreticulin. Calcium ions in green. (**A**) Calmodulin without calcium (well-structured), PDB_1CFC (**B**) Calcium-bound calmodulin, PDB_1UP5. The structure has changed, but remains well-ordered. (**C**) Calmodulin bound to a fragment of calcineurin PDB_2JZI. (**D**) Globular domain of calreticulin containing a single, high-affinity Ca^2+^ binding site, PDB_3POW.

Calmodulin (CaM) is a ubiquitous and highly conserved sensor that interacts with a wide variety of eukaryotic proteins and enzymes, controlling their activities in response to calcium [32,33]. Calmodulin is relatively small (16.7 kD) and has two globular domains, each containing a pair of EF-hand motifs, so in total it is capable of binding up to four calcium ions. Calcium binding induces significant conformational changes to CaM, promoting the binding of a wide range of protein factors. Such a variety of possible targets implies some degree of flexibility, but calmodulin, calcium-bound or not, it is very much ordered (apart from the flexible linker) (Figure 2A–C, NGL viewer [19]). It is observed that while calmodulin itself is not disordered, many of its protein targets display intrinsic disorder, which probably enable CaM’s promiscuity. Examples include the actin-binding protein caldesmon [34], PEP-19 (Purkinje cell protein 4, [35]), and the calcium-sensitive phosphatase, calcineurin. Additionally, bioinformatics was used to test the hypothesis that CaM-binding targets are intrinsically disordered [36]. Thus, disorder contributes to calcium signal transduction in the cytosol, though apparently, not directly.

One of the CaM targets is calcineurin (CaN), which is itself a calcium binding protein, but also displays a transient disorder [37]. This is, however, an exception, because most of the CaM targets do not bind to calcium, but exploit calmodulin as a sensor. The case of calcineurin is additionally baffling, because of the transient nature of the disorder. Apparently, as intracellular Ca^2+^ levels rise, CaN undergoes activation by both calcium and CaM binding. When CaN binds to Ca^2+^, one of its domains (the regulatory domain, RD) becomes disordered, and only in this state is it able to bind to calmodulin, which is consistent with the notion of disordered CaM targets. CaM binding induces RD folding, activating the phosphatase, so the disorder is transient, but crucial for the activity [37].

### 4.4. Calcium Sequestering Proteins of the Reticulum

The endoplasmic reticulum (ER) serves as the main calcium reservoir in the cell and the controlled release of Ca^2+^ from the ER regulates vital processes, like muscle contraction and signal transduction in neurons. The calcium storage proteins described here bind Ca^2+^ with high capacity, but low affinity, which is the typical mode of binding in IDPs. These multiple calcium-binding sites are located in the C-terminal, acidic, unstructured domain.

Calsequestrin, is a sarcoplamic reticulum protein of skeletal and cardiac muscle [38]. It binds 40–50 calcium ions per molecule. It has been shown that in calsequestrin’s negatively charged and disordered C-terminal, 27 amino acids take part in Ca^2+^ binding; its removal results in a 50% reduction in calcium binding properties [39].

Calreticulin, an endoplasmic reticulum protein, is not only a calcium-storage protein, but also acts as a molecular chaperone [40,41]. It has one high-affinity calcium-binding site within a globular domain (Figure 2D) and multiple low-affinity sites in its disordered C-terminal domain [42]. The C-terminal domain also contains an ER retention signal [41]. It was proposed that calreticulin acts as a calcium sensor in the ER lumen [40].

### 4.5. Other Calcium-Binding IDPs

Another interesting, yet isolated, example of a calcium-binding IDP is plant protein VCaB45. It belongs to a family of dehydrins: plant proteins, which are upregulated after abiotic stress, such as drought, freeze, or salinity [43]. They represent highly hydrophilic, intrinsically disordered proteins, with a basic, lysine-rich motif. Most dehydrins do not bind to Ca^2+^, though vacuolar VCaB45 is an exception. As in the case of Stm or DPP, the calcium-binding properties of dehydrin-like VCaB45 are modulated by phosphorylation; the phosphorylated protein binds up to 100-fold more calcium than the dephosphorylated protein [44].

The brief account of calcium-binding IDPs reviewed herein may be, and probably is, incomplete, because many relevant proteins may have been overlooked, representing cases of unreported disorder [45], especially if this disorder is transient. There also might be some cases of lesser known proteins, for which the calcium-binding properties have not yet been characterized, for instance, the predominantly mitochondrial protein HAX1, with predicted intrinsically disordered regions, where some preliminary studies have shown a capacity to bind to calcium [46] and also indicate a possible conformational change upon calcium binding [47].

## 5. Conclusions

As demonstrated in this review, calcium binding IDPs are mostly extracellular (in a folded form) or involved in secretion or calcium sequestering, and thus, they reside in locations in which Ca^2+^ concentration is relatively high. Binding of the divalent calcium ion has a great potential to force structural change, including protein folding, as shown here in the described examples. It is conceivable that after a calcium spike or wave, some cytosolic proteins may undergo folding and somehow propagate the message about the local increase in Ca^2+^ concentration. Why is this type of calcium sensing and signaling not common? Some possible answers are as follows:

- Ca^2+^ concentration in the cytoplasm is too low/calcium spike is too sharp and rapid (nevertheless such potential obstacles do not hinder calmodulin calcium-sensing).
- No secondary structure formed upon calcium binding in the cytoplasm can be as effective as the EF-hand motif (energetically unfavorable?).
- After Ca^2+^ binding and folding, how would the message be propagated?
- The Ca^2+^ dependent IDP sensor would have to bind too many protein targets; a universal calcium sensor (calmodulin) binding to many IDPs works better.
- There are presumably unknown calcium-binding IDPs.
- There are more examples of transient disorder; they may be difficult to spot and cannot be easily predicted from the sequence.

Whatever the reason for such a discrepancy in the IDPs’ role in the two main cellular signaling systems, possible causes appear to be deeply rooted in cell physiology and the fundamental laws of nature.

## Figures and Tables

**Table 1:** The best characterized calcium-binding proteins involved in biomineralization.

Protein Symbol	Protein Name	Organism	Function	Swiss Prot ID
OPN (SSP1)	Osteopontin, secreted phosphoprotein 1	*Homo sapiens*	Matrix mineralization, immunity	P10451
DMP1	Dentin matrix protein 1	*Homo sapiens*	Osteoblast differentiation, nucleation of hydroxyapatite	Q13316
MEPE	Matrix extracellular phosphoglycoprotein	*Homo sapiens*	Phosphate excretion, mineralization	Q9NQ76
BSP	Bone sialoprotein	*Homo sapiens*	Matrix mineralization, hydroxyapatite binding, cell attachment	P21815
DSPP	Dentin sialophosphoprotein	*Homo sapiens*	Dentinogenesis	Q9NZW4
AMELX	Amylogenin	*Homo sapiens*	Enamel formation; extracellular scaffold, crystal growth	Q99217
Stm	Starmaker	*Danio rerio* (zebrafish)	Otolith formation	A2VD23
OMM-64	Otolith matrix macromolecule-64	*Oncorhynchus mykiss* (rainbow trout)	Otolith formation	B1Q2L9
